# Exit interviews with caregivers of pediatric patients with classic galactosemia to explore meaningfulness of changes in the ACTION-galactosemia kids trial

**DOI:** 10.1186/s13023-025-04024-4

**Published:** 2025-09-30

**Authors:** Jason A. Randall, Carolyn Sutter, Stella Wang, Evan Bailey, Lydia Raither, Riccardo Perfetti, Shoshana Shendelman, Claire Burbridge

**Affiliations:** 1 Clinical Outcomes Solutions, Suite 8, Motis Business Centre Cheriton High Street, Folkestone, CT19 4QJ UK; 2 Clinical Outcomes Solutions, Chicago, IL USA; 3 Applied Therapeutics, New York, NY 10017 USA

**Keywords:** Classic galactosemia, Govorestat, Exit interview, Caregiver, Meaningful change

## Abstract

**Background:**

Classic Galactosemia is a rare, autosomal recessive disease in which galactose is not metabolized properly due to severe deficiency/absence of the galactose-1-phosphate uridylyltransferase (GALT) enzyme, converting to an aberrant and toxic metabolite, galactitol. Living with the debilitating symptoms and long-term consequences of Classic Galactosemia creates a heavy burden on patients’ and their families’ lives. Objectives were to: (1) Evaluate the impact and burden of disease; (2) Qualitatively explore changes in patient symptoms following treatment; and (3) Document the meaningfulness of changes resulting from treatment with govorestat as assessed by the Caregiver Global Impression of Severity (CGIS) and Caregiver Global Impression of Change (CGIC) scales.

**Methodology:**

The AT-007-1002 clinical trial involved a Phase 1/2 dose escalation component (Part A) followed by a Phase 3, randomized, double-blind, placebo-controlled long-term administration component (Part B) that evaluated potential clinical benefit. Exit Interviews were completed prior to unblinding of data. The in-depth, qualitative interviews were semi-structured, using a discussion guide, and conducted by either Zoom or GoToMeeting. Thirty-six caregiver interviews were conducted, capturing the experience of 37 pediatric patients (one caregiver had 2 patients enrolled in the study). Thematic analysis was undertaken to identify themes or patterns within the data. All analyses were conducted on blinded data. Following finalization of the analysis and report findings, post-hoc analysis of the unblinded data was then conducted to explore the meaningfulness of patients experience by treatment arm.

**Results:**

This study confirms the substantial burden known to be associated with Classic Galactosemia in a pediatric population. The difficulties experienced were across multiple areas including cognitive function, behavior/social function, motor function, emotional function, communication, vision problems, ovarian insufficiency, sensory difficulties, and sleep problems. The interviews demonstrated that most patients (approximately two thirds) experienced an improvement in symptoms and impacts associated with classic galactosemia over the course of the trial. Nearly all caregivers reported that they perceived a 1-category change on the Caregiver Global Impression of Severity or Caregiver Global Impression of Change items, indicating severity and change respectively, was meaningful to them and the patient. Unblinded analysis of the exit interview data confirmed the patient experience reported by caregivers was different between the treatment arms, providing qualitative support for the treatment benefit of govorestat when compared to placebo. Furthermore, the qualitative data from caregivers provide in-depth insights of their unique lived experience that highlight the substantial impact that this improvement had on the caregiver’s and the patient’s quality of life. The improvements observed led to a reduction on the burden of Classic Galactosemia and may lead to a greater patient’s greater independence.

**Conclusions:**

The exit interviews confirmed the burden of Classic Galactosemia disease across multiple domains. Qualitative investigation suggests that observed changes are meaningful to the patient and caregiver, and changes were more commonly reported in those receiving govorestat compared to placebo. Full clinical trial findings will be published separately.

## Background

Galactosemia is a rare autosomal recessive condition caused by mutations in the enzymes involved in the pathway of galactose metabolism. The most common form of galactosemia, known as Classic Galactosemia or Type I Galactosemia, is a result of a severe deficiency in the galactose-1-phosphate uridylyltransferase (GALT) enzyme that occurs in 1/16,000 to 1/60,000 births worldwide [1, 2].

If infants with Classic Galactosemia are not promptly started on a galactose-restricted diet, life-threatening complications appear within a few days after birth. While institution of a galactose-restricted diet generally prevents deaths, affected children may develop long-term progressive neurological complications, including behavioral issues, cognitive deficits, socialization problems, difficulty with activities of daily living, difficulties with adaptive skills, motor deficits, tremor and speech impairment, and may also develop cataracts and seizures [1, 2]. Long-term complications worsen in adults and most adults are unable to live independently [1, 3–7]. Female patients with Classic Galactosemia may develop premature ovarian insufficiency, and sterility [6, 7].

Living with the debilitating symptoms and long-term consequences of Classic Galactosemia creates a heavy burden on patients’ and their families’ lives [1, 4–9]. In 2 earlier studies, in adult and pediatric patients and their caregivers, we described the severe disease burden of Classic Galactosemia [8, 9]. As part of these studies, a conceptual model encompassing signs and symptoms, impacts, and health-related quality of life consequences associated with Classic Galactosemia was developed. This demonstrated that Classic Galactosemia inflicts a substantial deleterious impact on the patients’ health-related quality of life and a large unmet medical need remains [8, 9].

Applied Therapeutics conducted a Phase 1–3, sequential, 2-part study (AT-007-1002) that evaluated the clinical benefit, safety, pharmacokinetics and pharmacodynamics of govorestat (AT-007) (NCT04902781), first registration date: 14th July 2020. Govorestat is a once-daily oral compound that inhibits the enzyme aldose reductase, which plays a crucial role in the pathogenesis of galactosemia [10]. The primary objective of the AT-007-1002 clinical trial was to evaluate the clinical benefit of long-term orally administered govorestat on pediatric patients with Classic Galactosemia ages 2- through 17-years. This was evaluated using standardized clinical outcome assessments including several measures evaluating behavior and social function (behavioral function and adaptive behavior), cognitive function, motor function (balance/ataxia and dexterity), tremor, and communication. Treatment with govorestat provided sustained clinical benefit on activities of daily living, behavioral symptoms, cognition, adaptive behavior and tremor over 18 months of treatment [11]. An exit interview project was conducted as a pre-specified sub-study of the AT-007-1002 clinical trial and was designed to provide data to help contextualize and further understand the patient experience of Classic Galactosemia and of govorestat in a pediatric population.

Therefore, the objectives of this study were to: (1) Evaluate the impact and burden of the disease in this patient population, and to identify the most bothersome symptoms; (2) Qualitatively explore changes in the patient symptoms following the AT-007-1002 clinical trial; and (3) Document the meaningfulness of changes resulting from treatment with govorestat in the AT-007-1002 clinical trial as assessed by the Caregiver Global Impression of Severity (CGIS) and Caregiver Global Impression of Change (CGIC) scales.

## Methods

### Overview

The AT-007-1002 clinical trial involved a Phase 1/2 dose escalation component (Part A) followed by a Phase 3, randomized, double-blind, placebo-controlled long-term administration component (Part B) that evaluated potential clinical benefit. Clinical trial participants in Part B were randomized 2:1 to receive either AT-007 (optimum dose) or placebo. The duration of treatment in Part B was not predefined; the Part B continued until there was substantial evidence of clinical benefit (or futility), as determined by an unblinded firewalled Data Monitoring Committee, which evaluated unblinded data every 6 months. The decision that evidence of clinical benefit had been reached was made after the 3rd evaluation of the Data Monitoring Committee in April 2023 (i.e., after 18 months). Clinical trial participants who had been randomized to placebo could continue on to an open label extension study and those who had been randomized to govorestat treatment could enroll in an expanded access program.

The conduct of exit interviews was pre-specified in the protocol and was completed prior to unblinding of patients, caregivers, and clinical sites. They were performed by an independent, blinded third party who are specialists in conducting qualitative research. The in-depth, qualitative interviews were semi-structured, using a discussion guide, and conducted by either Zoom or GoToMeeting.

### Ethical approval and consent

Institutional Review Board approval was obtained from Western-Copernicus Group (tracking number: 20211752). All participants were required to sign and date the informed consent form before conducting study activities, which also included permission to published anonymized data and quotes. All participants were also reminded that they could withdraw their consent at any time without compromising their clinical care and they would still receive participation compensation for tasks they had completed. All data collected and processed for this study were managed by an independent company with adequate precautions to ensure the confidentiality of the data and following applicable national and/or local laws and regulations on personal data protection.

### Interview conduct

The qualitative researchers who designed and conducted this study are from an organization impartial and independent independent of the clinical trial. The clinical trial team were not involved in the conduct of the interviews and the interviewers were impartial. The researchers are experience in qualitative research, with training and substantial qualitative field experience in the conduct of studies to understand and explore patient experience.

All interviews were conducted by an impartial organization, independent of the clinical trial. All interviewers were experienced in qualitative research with training from academic degrees, and substantial qualitative experience in the field. All interviewers also received training on the medical condition, and the discussion guide to ensure that they would understand the patient voice and any medical terms that might be used by the caregivers being interviewed.

Forty of the 47 patients enrolled completed the trial through 18-months. A total of 38 exit interviews were conducted with the caregivers of pediatric patients which represented all 40 patients who completed the clinical trial (2 caregivers represented 2 patients within the clinical trial each). Unfortunately, two audio files became corrupted and could not be sent for transcription. Therefore, transcripts from 36 interviews were analyzed. These 36 interviews represented 37 pediatric patients (as the audio from 1 of the caregivers representing 2 patients in the trial was corrupted), which is 95% of the patients who completed the trial through 18 months.

To reduce any potential bias, it was intended to conduct as many interviews as possible before the decision to stop the trial became public (the public announcement was scheduled for the week after the decision was made). A total of 34 interviews occurred over the weekend immediately following decision to end trial (before public announcement of the trial ending) and 4 interviews occurred the following week (following the announcement). Four experienced, trained interviewers were used to support this tight timeline.

During interviews, caregivers were asked to describe the patient’s experience of Classic Galactosemia before the trial and to describe any changes observed during the trial. Caregivers were asked whether these changes (or lack of change) were meaningful to both the patient and the caregiver and why. Caregivers were also asked to discuss change in relation to 2 global assessment items that were used in the clinical trial: CGIS (disease severity) or CGIC).

### Analysis

Interviews were audio-recorded and transcribed verbatim. De-identified transcripts were uploaded to NVivo, a qualitative analysis software program. Thematic analysis was undertaken by 2 expert researchers who coded the data to identify any themes being discussed and patterns emerging from the data. A codebook was developed by the study team to ensure that all codes were similar in style, as well as to clarify how codes should be applied. The coding was reviewed and discussed with the study lead throughout to ensure codes were being applied consistently and appropriately. All initial analyses and reporting were conducted on blinded data. Post-hoc comparisons using unblinded data to explore any differences by treatment arm were then conducted; this was initiated only after full analysis and reporting was complete. Analysis and interpretation of the interview data was conducted by qualitative researchers independent of the sponsor. The sponsor was not involved in the coding of the data and did not have access to raw data.

## Results

### Sample characteristics

Within the 37 pediatric patients represented in the interview analysis, the mean age was 9.4 years (range from 2- to 16-years) and there was similar representation across the targeted age groups (*n* = 12, 14 and 11, respectively, across the age groups ≥ 2 to ≤ 6-years, *≥* 7 to ≤ 12-years, and ≥ 13 to < 18-years). There were slightly more female patients (54.1%) than male; this was consistent across the age groups. The majority of patients were white (97.3%), not Hispanic or Latino (97.3%). A little under half of patients’ GALT gene mutations were homozygous for Q188R allele (43.2%), which is the most common mutation and results in complete loss of enzyme activity. All patients had < 1% GALT enzyme activity (median 0.0 nmol/h/mg of hemoglobin) which is diagnostic for Classic Galactosemia. The full demographics of the patients can be found in Table 1.

**Table 1:** Demographic and clinical characteristics of patients represented in the interviews

Patient Demographic or Clinical Parameter		Patients 2–6 Years (*n* = 12)	Patients 7–12 Years (*n* = 14)	Patients 13–18 Years (*n* = 11)	Total(*N* = 37)
	n*	12	14	11	37
	Female	7 (58.3%)	7 (50.0%)	6 (54.5%)	20 (54.1%)
	Male	5 (41.7%)	7 (50.0%)	5 (45.5%)	17 (45.9%)
	Prefer not to answer	0 (0%)	0 (0%)	0 (0%)	0 (0%)
	n missing	0	0	0	0
	n	12	14	11	37
	Mean (SD)	4.6 (1.44)	9.5 (1.61)	14.4 (1.43)	9.4 (4.17)
	Q1	3.5	8.0	13.0	6.0
	Median	4.5	10.0	14.0	10.0
	Q3	6.0	10.0	16.0	13.0
	Min, Max	2, 6	7, 12	13, 16	2, 16
	n missing	0	0	0	0
Ethnicity, n (%)	n*	12	14	11	37
	Hispanic/Latino	0 (0%)	1 (7.1%)	0 (0%)	1 (2.7%)
	Not Hispanic/Latino	12 (100%)	13 (92.9%)	11 (100%)	36 (97.3%)
	n missing	0	0	0	0
Race, n (%)	White	12 (100%)	13 (92.9%)	11 (100%)	36 (97.3%)
	Black/African American	0 (0%)	0 (0%)	0 (0%)	0 (0%)
	American Indian/Alaska Native	0 (0%)	0 (0%)	0 (0%)	0 (0%)
	Asian/Asian American	0 (0%)	0 (0%)	0 (0%)	0 (0%)
	Native Hawaiian/Pacific Islander	0 (0%)	0 (0%)	0 (0%)	0 (0%)
	Other: Hispanic	0 (0%)	1 (7.1%)	0 (0%)	1 (2.7%)
GALT Gene Mutations, n (%)	n*	12	14	11	37
	Homozygous Q188R	5 (41.7%)	6 (42.9%)	5 (45.5%)	16 (43.2%)
	Compound Heterozygous Q188R^[b]^	6 (50.0%)	6 (42.9%)	5 (45.5%)	17 (45.9%)
	Other Compound Heterozygous^[b]^	1 (8.3%)	2 (14.3%)	1 (9.1%)	4 (10.8%)
	n missing	0	0	0	0
GALT Enzyme Activity, n (%)	n*	11	14	11	36
	Mean (SD)	0.00 (0.000)	0.02 (0.043)	0.02 (0.040)	0.01 (0.035)
	Q1	0.00	0.00	0.00	0.00
	Median	0.00	0.00	0.00	0.00
	Q3	0.00	0.00	0.00	0.00
	Min, Max	0.0, 0.0	0.0, 0.1	0.0, 0.1	0.0, 0.1
	n missing	1	0	0	1

*GALT* gal-1-P uridylyl transferase, *max* maximum, *min* minimum, *n* number of subjects in the population, *Q1* 25th percentile, *Q3* 75th percentile, *SD* standard deviation

*Number of subjects with non-missing data, used as the denominator

^a^Age is the Age at Trial Entry

^b^The non-Q188R compound heterozygous mutations include A320V, C.253–2 A > G, Del Exons 1–11, E203K, F171LFS*7, I278N, K285N, L195P, M142K, P36L, Q212, Q334K, Q353, R201H, R204, R272C, S135L, Y209C, Y251S and Other. Other refers to a mutation that is not part of the panel of the 14 most common GALT gene mutations

All caregivers who took part in the interviews were either the parent or stepparent of the patient who participated in the clinical trial. The majority of caregivers were female (36/37; 97.2%) and were aged from 31- to 53-years old at the time of the exit interview (average age 42-years old). The majority of caregivers were white (97.2%) and not Hispanic or Latino (97.2%). Most caregivers had completed a college degree (55.6%) or graduate degree (22.2%). Most caregivers (61.1%) were employed full-time, a quarter (25%) identified as a homemaker, and the remaining caregivers were employed part-time. The full demographics of the caregivers can be found in Table 2.

**Table 2:** Caregiver demographic characteristics

Caregiver Demographic Parameter		Patients 2–6 Years (*n* = 12)	Patients 7–12 Years (*n* = 13)	Patients 13–18 Years (*n* = 11) ^a^	Total (*N* = 36)
Gender, n (%)	n*	12	13	11	36
	Female	11 (91.7%)	13 (100%)	11 (100%)	35 (97.2%)
	Male	1 (8.3%)	0 (0%)	0 (0%)	1 (2.8%)
	Prefer not to answer	0 (0%)	0 (0%)	0 (0%)	0 (0%)
	n missing	0	0	0	0
Age (Years)	n	12	13	10	35
	Mean (SD)	37.7 (5.02)	42.1 (4.66)	45.2 (4.54)	41.5 (5.53)
	Q1	34.0	38.0	41.0	37.0
	Median	36.0	42.0	47.0	42.0
	Q3	42.0	44.0	48.0	46.0
	Min, Max	31, 47	36, 53	37, 52	31, 53
	n missing	0	0	1	1
Ethnicity, n (%)	n*	12	13	11	36
	Hispanic/Latino	0 (0%)	1 (7.7%)	0 (0%)	1 (2.8%)
	Not Hispanic/Latino	12 (100%)	12 (92.3%)	11 (100%)	35 (97.2%)
	n missing	0	0	0	0
Race, n (%)	White/Caucasian	12 (100%)	13 (100%)	10 (90.9%)	35 (97.2%)
	Black/African American	0 (0%)	0 (0%)	0 (0%)	0 (0%)
	American Indian/Alaska Native	0 (0%)	0 (0%)	0 (0%)	0 (0%)
	Asian/Asian American	0 (0%)	0 (0%)	0 (0%)	0 (0%)
	Native Hawaiian/Pacific Islander	0 (0%)	0 (0%)	0 (0%)	0 (0%)
	Other	0 (0%)	0 (0%)	1 (9.1%)	1 (2.8%)
Relationship to Patient, n (%)	n*	12	13	11	36
	Parent or Stepparent	12 (100%)	13 (100%)	11 (100%)	36 (100%)
	Aunt/Uncle	0 (0%)	0 (0%)	0 (0%)	0 (0%)
	Grandparent	0 (0%)	0 (0%)	0 (0%)	0 (0%)
	Older Sibling	0 (0%)	0 (0%)	0 (0%)	0 (0%)
	Legal Guardian or Caretaker	0 (0%)	0 (0%)	0 (0%)	0 (0%)
	Foster parent	0 (0%)	0 (0%)	0 (0%)	0 (0%)
	Other	0 (0%)	0 (0%)	0 (0%)	0 (0%)
	n missing	0	0	0	0
Education, n (%)	n*	12	13	11	36
	Did not complete high school	0 (0%)	0 (0%)	0 (0%)	0 (0%)
	High school diploma or General Education Diploma	0 (0%)	2 (15.4%)	0 (0%)	2 (5.6%)
	Some college/ or certification program	1 (8.3%)	3 (23.1%)	2 (18.2%)	6 (16.7%)
	College, technical college, or university degree (2- or 4- year)	7 (58.3%)	6 (46.2%)	7 (63.6%)	20 (55.6%)
	Graduate degree (PhD, MD, etc.)	4 (33.3%)	2 (15.4%)	2 (18.2%)	8 (22.2%)
	Other	0 (0%)	0 (0%)	0 (0%)	0 (0%)
	n missing	0	0	0	0
Work Status, n (%)	n*	12	13	11	36
	Employed full-time/self-employed (≥ 40 h per week)	6 (50.0%)	7 (53.8%)	9 (81.8%)	22 (61.1%)
	Employed part-time/self-employed (< 40 h per week)	0 (0%)	4 (30.8%)	1 (9.1%)	5 (13.9%)
	Homemaker	6 (50.0%)	2 (15.4%)	1 (9.1%)	9 (25.0%)
	Not working due to a disability	0 (0%)	0 (0%)	0 (0%)	0 (0%)
	Student	0 (0%)	0 (0%)	0 (0%)	0 (0%)
	Retired	0 (0%)	0 (0%)	0 (0%)	0 (0%)
	Unemployed	0 (0%)	0 (0%)	0 (0%)	0 (0%)
	n missing	0	0	0	0

*Max* maximum, *min* minimum, *n* number of subjects in the population, *Q1* 25th percentile, *Q3* 75th percentile, *SD* standard deviation

*Number of subjects with non-missing data, used as the denominator

^a^The caregiver interviewed who discussed 2 patients they cared for that participated in the clinical trial is included in the data for the patient in the oldest age group (13–18 years), although they also discussed their child in the middle age group (7–12 years)

### Lived experience prior to the trial (Blinded data Analysis)

Prior to the trial, caregivers reported that patients experienced difficulties within multiple areas consistent with the known burden of disease in Classic Galactosemia (see Table 3). The symptoms and impacts most commonly reported were cognitive function (92%), motor function (84%), communication (76%), emotional function (73%), and behavior/social function (65%). Caregivers also described patients’ experience of vision problems, ovarian insufficiency (for females), sensory difficulties, and sleep problems. Most symptoms and impacts were described spontaneously by caregivers with some endorsing after probing; vision problems however were only described after probing. There were no obvious differences between age groups regarding the overall domains that were reported to be impacted by Classic Galactosemia (see Table 3), although the specific areas impacted within the domains reflected activities or functions that were age appropriate e.g., cognitive function abilities that were impacted were appropriate based on the child’s age Tables 4, 5

**Table 3:** Classic galactosemia difficulties/burden described before the clinical trial

Difficulties/ burden	Patients 2–6 Years (*n* = 12)				Patients 7–12 Years (*n* = 14)			Patients 13–18 Years (*n* = 11)			Full Sample(*N* = 37)		
	Total	S	*P*		Total	S	*P*	Total	S	*P*	Total	S	*P*
Cognitive difficulties	10/12 (83%)	9/10	1/10		14/14 (100%)	11/14	3/14	10/11 (91%)	10/10	0/10	34/37 (92%)	30/34	4/34
Motor difficulties	10/12 (83%)	6/10	4/10		11/14 (79%)	5/11	6/11	10/11 (91%)	4/10	6/10	31/37 (84%)	15/31	16/31
Communication difficulties	10/12 (83%)	8/10	2/10		11/14 (79%)	8/11	3/11	7/11 (64%)	4/7	3/7	28/37 (76%)	20/28	8/28
Emotional difficulties	8/12 (67%)	4/8	4/8		11/14 (79%)	7/11	4/11	8/11 (73%)	4/8	4/8	27/37 (73%)	15/27	12/27
Behavior/ Social difficulties	7/12 (58%)	6/7	1/7		10/14 (71%)	6/10	4/10	7/11 (64%)	3/7	4/7	24/37 (65%)	15/24	9/24
Vision problems^a^	1/12 (8%)	0/1	1/1		4/14 (29%)	0/4	4/4	7/11 (64%)	0/7	7/7	12/37 (32%)	0/12	12/12
Ovarian insufficiency^b^	1/7 (14%)	1/1	0/1		4/7 (57%)	2/4	2/4	5/6 (83%)	1/5	4/5	10/20 (50%)	3/10	7/10
Sensory difficulties^b^	4/12 (33%)	4/4	0/4		2/14 (14%)	2/2	0/2	1/11 (9%)	1/1	0/1	7/37 (19%)	7/7	0/7
Sleep problems^d^	1/12 (8%)	1/1	0/1		0/14 (0%)	--	--	2/11 (18%)	2/2	0/2	3/37 (8%)	3/3	0/3

Difficulties discussed by only 1 or 2 caregivers were not included as they were deemed to be idiosyncratic and not add to the key value messages; these included constipated bowel, vomiting, joint pain, kyphosis of the spine, and short stature described by 1 caregiver each and seizures described by 2 caregivers

*P* described after interviewer probed, *S* caregivers spontaneously discussed

^a^Caregivers described cataracts (*n* = 2), a known complication of Classic Galactosemia, as well as need to wear glasses (*n* = 10) which is not part of the condition

^b^Ovarian insufficiency only reported for patients identified as female and so the percentage shown is out of the female population in each group

^c^Sensory difficulties were described by caregivers as: sensory integration disorder, sensory processing disorder, impacts on sensory system, and auditory processing difficulties

^d^Sleep problems described by caregivers included: struggling to fall asleep and to stay asleep

**Table 4:** Qualitative discussion of change in classic galactosemia symptoms and impacts

Symptoms and Impacts	Change in Qualitative Discussion	Patients 2–6 Years (*n* = 12)		Patients 7–12 Years (*n* = 14)		Patients 13–18 Years (*n* = 11)		Full Sample (*N* = 37)	
		Total	Was the Change Meaningful?^**a**^	Total	Was the Change Meaningful?^a^	Total	Was the Change Meaningful?^a^	Total	Was the Change Meaningful?^a^
Cognitive Difficulties	Improvement	7/10 (70%)	6/7 (86%)	8/14 (57%)	8/8 (100%)	8/10 (80%)	8/8 (100%)	23/34 (68%)	22/23 (96%)
	No change	1/10 (10%)	N/A^b^	6/14 (43%)	3/4 (75%)^b^	2/10 (20%)	1/2 (50%)	9/34 (26%)	4/6 (67%)^b^
	Worsened^c^	2/10 (20%)	1/1 (100%)^b^	0/14 (0%)	N/A	0/10 (0%)	N/A	2/34 (6%)	1/1 (100%)^b^
Motor Difficulties	Improvement	7/10 (70%)	7/7(100%)	6/11 (55%)	6/6 (100%)	4/10 (40%)	4/4 (100%)	17/31 (55%)	17/17 (100%)
	No change	3/10 (30%)	2/3 (67%)^b^	4/11 (36%)	2/3 (67%)	6/10 (60%)	1/3 (33%)^b^	13/31 (42%)	5/9 (56%)^b^
	Worsened^c^	0/9 (0%)	N/A	1/11 (9%)	1/1 (100%)	0/10 (0%)	N/A	1/31 (3%)	1/1 (100%)
Communication Difficulties	Improvement	8/10 (80%)	8/8 (100%)	9/11 (82%)	9/9 (100%)	5/7 (71%)	5/5 (100%)	22/28 (79%)	22/22 (100%)
	No change	2/10 (20%)	2/2 (100%)	2/11 (18%)	0/1 (0%)^b^	2/7 (29%)	1/1 (100%)^b^	6/28 (21%)	3/4 (75%)^b^
	Worsened^c^	0/10 (0%)	N/A	0/11 (0%)	N/A	0/7 (0%)	N/A	0/28 (0%)	N/A
Emotional difficulties	Improvement	6/8 (75%)	6/6 (100%)	5/11 (45%)	5/5 (100%)	3/8 (38%)	3/3 (100%)	14/27 (52%)	14/14 (100%)
	No change	0/8 (0%)	N/A	4/11 (36%)	2/2 (100%)^b^	4/8 (50%)	1/2 (50%)^b^	8/27 (30%)	3/4 (75%)^b^
	Worsened^c^	2/8 (25%)	2/2 (100%)	2/11 (18%)	2/2 (100%)	1/8 (12%)	1/1 (100%)	5/27 (19%)	5/5 (100%)
Behavior/ Social difficulties	Improvement	6/7 (86%)	6/6 (100%)	5/10 (50%)	5/5 (100%)	5/7 (71%)	5/5 (100%)	16/24 (67%)	16/16 (100%)
	No change	0/6 (0%)	N/A	4/10 (40%)	2/3 (67%)^b^	2/7 (29%)	0/1 (0%)^b^	6/24 (25%)	2/4 (50%)^b^
	Worsened^c^	1/7 (14%)	1/1 (100%)	1/10 (10%)	1/1 (100%)	0/7 (0%)	N/A	2/24 (8%)	2/2 (100%)

^a^These values represent caregivers’ descriptions of meaningful change as part of the qualitative discussion, not related to the global assessment

^b^In some instances, the caregiver was not pushed to discuss whether a change was meaningful, particularly for those who had seen no change or a worsening, particularly if this was uncomfortable for the caregiver

^c^For difficulties reported to worsen caregivers were asked if this worsening was meaningful

**Table 5:** Qualitative discussion of change in other biological symptoms

Symptoms and Impacts	Change in Qualitative Discussion	Patients 2–6 Years (*n* = 12)		Patients 7–12 Years (*n* = 14)		Patients 13–18 Years (*n* = 11)		Full Sample (*N* = 37)	
		Total	Was the Change Meaningful?^a^	Total	Was the Change Meaningful?^a^	Total	Was the Change Meaningful?^a^	Total	Was the Change Meaningful?^a^
Vision problems	Improvement	0/1 (0%)	N/A	0/4 (0%)	N/A	0/7 (0%)	N/A	0/12 (0%)	N/A
	No change	0/1 (0%)	N/A	4/4 (100%)	0/1 (0%)^b^	5/7 (71%)	1/3 (33%)^b^	9/12 (75%)	1/4 (25%)^b^
	Worsened^c^	1/1 (100%)	1/1 (100%)	0/4 (0%)	N/A	2/7 (29%)	0/1 (0%)^b^	3/12 (25%)	1/2 (50%)^b^
Ovarian insufficiency^d^	Improvement	0/1 (0%)	N/A	2/4 (50%)	2/2 (100%)	1/5 (20%)	1/1 (100%)	3/10 (30%)	3/3 (100%)
	No change	1/1 (100%)	N/A^b^	2/4 (50%)	N/A^b^	3/5 (60%)	1/1 (100%)^b^	6/10 (60%)	1/1 (100%)^b^
	Worsened^c^	0/1 (0%)	N/A	0/4 (0%)	N/A	1/5 (20%)	1/1 (100%)	1/10 (10%)	1/1 (100%)
Sensory difficulties	Improvement	3/4 (75%)	3/3 (100%)	1/2 (50%)	N/A^b^	0/1 (0%)	N/A	4/7 (57%)	3/3 (100%)^b^
	No change	0/4 (0%)	N/A	1/2 (50%)	1/1 (100%)	1/1 (100%)	N/A^b^	2/7 (29%)	1/1 (100%)^b^
	Worsened^c^	1/4 (25%)	1/1 (100%)	0/2 (50%)	N/A	0/1 (0%)	N/A	1/7 (14%)	1/1 (100%)
Sleep problems	Improvement	1/1 (100%)	1/1 (100%)	N/A	N/A	1/2 (50%)	1/1 (100%)	2/3 (67%)	2/2 (100%)
	No change	0/1 (0%)	N/A	N/A	N/A	1/2 (50%)	1/1 (100%)	1/3 (33%)	1/1 (100%)
	Worsened^c^	0/1 (0%)	N/A	N/A	N/A	0/2 (0%)	N/A	0/3 (0%)	N/A

^a^These values represent caregivers’ descriptions of meaningful change as part of the qualitative discussion, not related to the global assessment

^b^In some instances, the caregiver was not pushed to discuss whether a change was meaningful, particularly for those who had seen no change or a worsening, particularly if this was uncomfortable for the caregiver

^c^For difficulties reported to worsen caregivers were asked if this worsening was meaningful

^d^Ovarian insufficiency only reported for patients identified as female

Caregivers describing patients’ cognitive difficulties focused on short- and long-term memory, slow processing, difficulty following directions, challenges with attention, and overall cognitive developmental delays: “*it [cognitive function difficulties] was pretty severe. She would learn something and*,* 30 minutes later*,* it’s like she didn’t learn it… Um*,* or even she would know something for several weeks and then not know it anymore*” (2005-208-20, patient aged 7–12). Patients also had difficulties related to motor function such as handwriting, fine motor skills, and tremor which often involved muscle weakness and low muscle tone. The difficulties experienced were generally consistent across age groups, although motor function related to muscle weakness or low muscle tone was mainly described by caregivers of the youngest patients (2–6 years): “*Before*,* it was like*,* uh*,* every time she woke up*,* she would*,* like*,* have tremor*,* or when she was doing handwriting*,* like*,* purposeful movements*,* she would tremor*” (2005-309-20, patient aged 2–6 years).

Caregivers also discussed how patients had difficulties with communication and discussed how they saw issues with pronunciation, general communication difficulties, apraxia, and vocabulary difficulties: “*His*,* um*,* articulation. Um*,* he wasn’t able to be understood. Um-… his grammar*,* his vocabulary were very*,* uh*,* his vocabulary was very limited. . He was definitely severely impacted by*,* um*,* his speech. He was very*,* severely impaired”* (2005-212-20, patient aged 7–12 years). These difficulties were reported consistently across all patient age groups,

Based on the difficulties experienced, caregivers discussed how the child would experience emotional difficulties such as anxiety, low mood, and deviant behavior.

Overall, emotional difficulties were reported more frequently by caregivers of younger age children (2–6 years and 7–12 years). This was particularly related to what caregivers referred to as defiant behavior and emotional “control” (especially in children aged 2–6 years and 7–12 years) and focused on emotional outbursts, aggression, and the child being upset: *“Well*,* like*,* having an upset child all the time*,* is*,* like*,* super stressful*,* and contributes to caregiver burnout. Because you can’t… solve the problem”* (2005-309-20, patient aged 2–6 years). In comparison, caregiver reports of patient anxiety was consistent across all age groups, whilst feelings of low mood and depression, were only reported by caregivers of older patients (13–18 years).

Related to cognitive and motor difficulties, caregivers described that may experience social and behavioral issues, difficulty making or maintaining friendships, difficulty with social skills and difficulty experiencing new situations: *“She had a hard time with making friends because*,* um*,* you know*,* kids were trying to talk to her and she just wasn’t quick enough or they couldn’t… she couldn’t explain herself or carry on conversations at the level they could”* (2005-217-20, patient aged 7–12 years).

### Changes experienced over course of trial (Blinded data Analysis)

Overall, cognitive functioning was the area most commonly described as being improved over the course of the trial with caregivers highlighting memory improvement, enhanced information processing and speed, better ability to follow instructions, better focusing, and better retention of information: “*Um*,* we don’t have this problem [memory] anymore*,* she is able to keep up*,* she retains information so much better. We don’t have to constantly repeat the same thing over and over again. When she’s in school*,* she’s able to follow multi-step processes that she previously couldn’t do*” (2005-201-10, patient aged 7–12 years). Caregivers of patients who experience cognitive difficulties, 71% (24/34) reported improvement in these cognitive difficulties. This was consistent for patients of all age groups. These cognitive improvements had a significant impact on patients’ day-to-day lives which were also noticeable to others: “*her teacher really has even told me. She said*,* [Caregiver Name]*,* if someone would walk in here now*,* they wouldn’t even know that [Child Name] has galactosemia*” (2005-310-20, patient aged 2–6 years).

As part of the trial, caregivers also described how motor function in /the child improved, related to the patients’ fine motor skills, better ability to grip objectives, handwriting, and tremor. This was particularly the case for patients in the youngest age group (2–6 years): “*Her occupational therapist*,* her general education teacher*,* and her special education teacher have all made comments to me*,* um*,* not at the same time*,* and they didn’t even know she was in the drug trial*,* but within one to three months after being on the drug*,* they were all telling me like*,* “Wow*,* [Child Name]’s handwriting has improved…her handwriting*,* her legibility have improved”*” (2005-310-20, patient aged 2–6 years). Of those patients who experience motor function difficulties, 55% experienced improvement as part of the trial (17/31).

Similarly, caregivers (22/28) observed and described improvements in 79% of the children, regarding ability to form words, speak more quickly, and to overall communicate more easily without forgetting words or struggling to find the right words : “*improving*,* just*,* like*,* like*,* over that summer*,* like*,* just becoming a very talkative kid. And it’s something-… that blows my mind*,* because it was just*,* like*,* she wasn’t like that before… And now*,* she is telling me she’s trying to find monsters in the hallway*,* or*,* you know*,* she’s telling me a make-believe story*” (2005-309-20, patient aged 2–6 years). For some caregivers, improvements such as in pronunciation meant that the pre-trial challenges they faced regarding understanding the patient and the associated frustration had lessened: “*I don’t have trouble understanding what he’s trying to say to me [now]*” (2005-205-10, patient aged 7–12 years) which resulted in less emotional impacts and burden on the caregiver and family members.

Caregivers also observed improvements in patient’s social and behavioral difficulties, and described how the child interacted better with others, made new friends more easily, had increased play with others, and was better able to connect with their peers. Caregivers of patients in the youngest age group discussed the patients’ improved ability to play and connect with peers, in relation to their communication improvements, “*she had a play date at the house*,* and I couldn’t believe the back and forth conversation that was happening*” (2005-309-20, patient aged 2–6 years). Caregivers of older patients described changes in terms of ability to make friends, also often associated with communication. Of the patients who experienced these difficulties before the trial, caregivers of 67% of the patients stated that they had seen an improvement in these difficulties (16/24). Improvements were described across all social and behavioral difficulties.

Similarly, as these other difficulties were improving so were the patients’ emotional difficulties. Of the patients who experienced emotional difficulties before the trial, 52% of caregivers reported how they had seen an improvement in these during the trial (14/27, 52%). Based on the difficulties reported before the trial, there were some differences between age groups. Caregivers of patients in the youngest age group most frequently described improvements in terms of the patient’s ability to regulate their emotions, leading to fewer tantrums or meltdowns, whilst caregivers in the older age groups were more likely to see improvements in feelings of low mood. Caregivers explained how this led to the patient being happier: “*Um*,* but I feel like overall*,* she’s been a happier child*,* um*,* and people tell me that like*,* ‘[Child Name]’s so happy.’ Um*,* and you know*,* I… Again*,* I would say within one to three months after… I mean*,* it’s hard to pinpoint a day*,* but started seeing a improvement in her overall mood. Um*,* and then that has continued*” (2005-310-20, patient aged 2–6 years). Caregivers explained that improvements in anxiety meant that their lives and the patient’s life were not as disrupted: “*Yes [the improvement is meaningful]*,* he went from this [anxiety] being something that could disrupt his life and impact his quality of life*,* to just something that we- we can manage*,* and- and live normally*” (2005-313-20, patient aged 2–6 years).

### Meaningful change on global assessments (Blinded data Analysis)

When discussing the CGIS, the qualitative findings suggest that a 1-category change on the CGIS is meaningful. This was discussed by 28/37 caregivers, although some caregivers were not able to address this due to time constraints in the interview. For those caregivers who discussed this, all except 1 indicated that a 1-category change on the CGIS in all domains, with even small improvements would be meaningful to both them and the patient. Those caregivers who indicated that a 1-category change was meaningful discussed that any improvements, no matter how small, would always be meaningful to them as caregiver and the patient. The one remaining caregiver who discussed improvements said that a 1-category change would be meaningful for all domains except motor difficulties, where they would like to see a 2-category change.

When discussing the CGIC, all caregivers asked identified that any change (i.e., “A little better” or “Much better” on the CGIC) represented a meaningful change to both caregiver and the patient. This was discussed by 28/37 caregivers due to time constraints in the interview. All except 1 caregiver reported that a “A little better” was meaningful for all domains, meaningful change (27/28) except for motor function. For motor function the majority of caregivers (24/28) reported that any change would be meaningful; the 4 who did not, indicated “A little better” for motor function was not meaningful because they were hoping for a larger change of “Much better.”

Additionally, when discussing both the CGIS and CGIC items, caregivers noted that not only an improvement would be meaningful, but for there not to be any worsening as this indicated that the patient’s condition has not progressed: “*it’s meaningful that it hasn’t got worse… uh*,* which I think does happen with some kids. So*,* you know*,* it’s meaningful to me that it’s not gotten worse”* (2005-303-10, patient aged 2–6 years).

### Comparison of findings by treatment arm (Unblinded analysis)

**Fig. 1:**
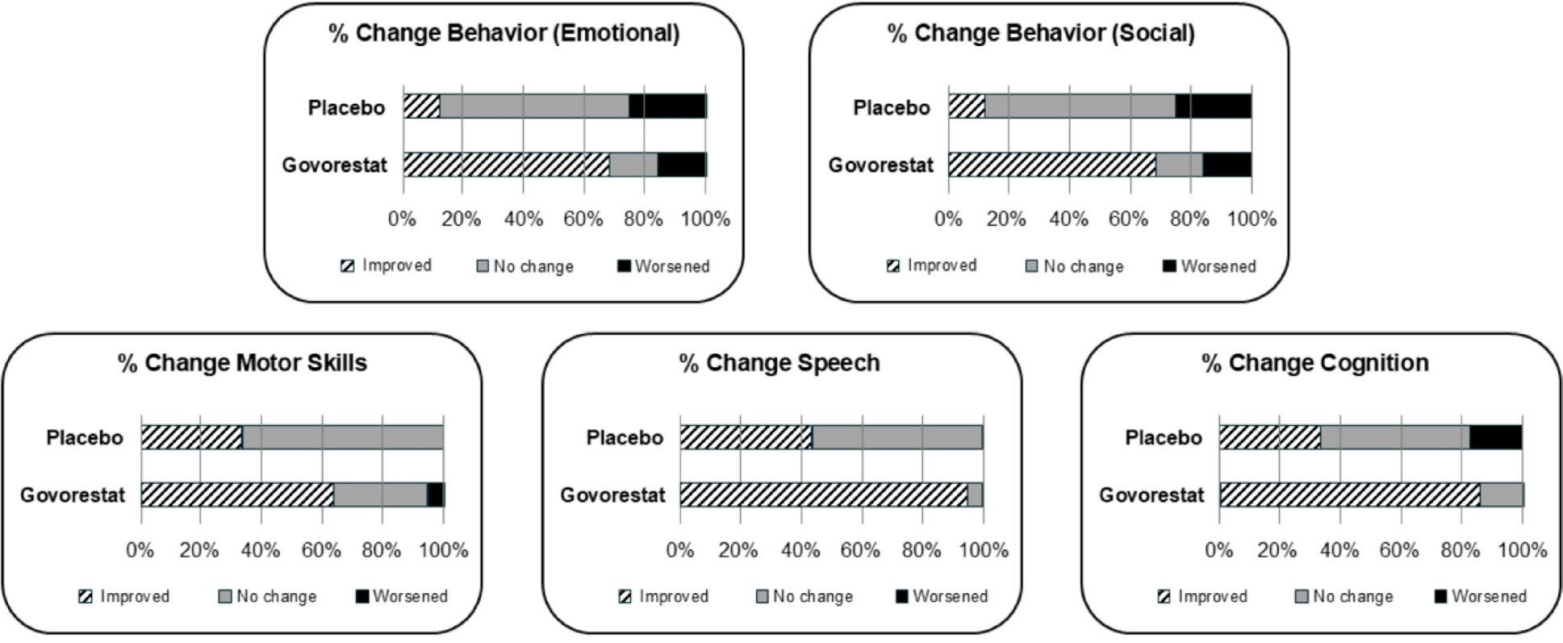
Improvement by treatment arm

Unblinded analysis compared pre- and post-trial responses from caregivers whose child experienced difficulties at baseline. When the unblinded analysis was conducted, the comparison of qualitative feedback across treatment arms highlighted those caregivers of patients randomized to govorestat treatment reported more favorable outcomes compared to the caregivers of patients randomized to placebo. This was consistent for all concepts reported as being experienced by the patient, see Fig. 1. In the govorestat group, 86.4% of patients reported an improvement in cognition, while only 33.3% did so in the placebo group. Similarly for patients who experienced motor difficulties 63.6% of patients treated with govorestat reported improvement in motor symptoms as compared to 33.3% in the placebo group. Similarly, in the govorestat group 94.7% (18/19) reported improvement in speech compared to 44.4% (4/9) in the placebo group. For those who experienced emotional difficulties in the govorestat group, 68.4% reported improvement in emotional difficulties compared to 12.5% in the placebo group. Behavioral or social difficulties improved for 76.4% (13/17) of the patients in the govorestat group compared to 42.9% (3/7) in the placebo group.

## Discussion

Findings from this exit interview substudy provide rich, qualitative data giving insight into patients’ and caregivers’ experiences with Classic Galactosemia and with the clinical trial of govorestat. The interviews gave the caregivers the opportunity to share their observations of changes in the patient’s daily function and challenges associated with Classic Galactosemia and their perceptions of what these changes meant to them and to the patient.

This study confirms the substantial burden known to be associated with Classic Galactosemia in a pediatric population. The difficulties that patients experienced due to Classic Galactosemia were across multiple areas including cognitive function, behavior/social function, motor function, emotional function, and communication. Caregivers also described patients’ experience of vision problems, ovarian insufficiency (for females), sensory difficulties, and sleep problems. Caregiver descriptions of the patient experience before the trial align with the literature and previous qualitative research, which also demonstrated the burden of Classic Galactosemia across the forementioned areas.^8,9^ This research therefore provides further evidence of the high unmet medical need in this condition, the need for further support for patients and caregivers, and the need for a treatment.

The interviews also demonstrated that, as perceived and reported by the caregivers, most patients (approximately two thirds) experienced an improvement in symptoms and impacts associated with Classic Galactosemia over the course of the AT-007 clinical trial. Moreover, the blinded exit interview data was further confirmed through unblinded analysis of the data. Caregivers typically felt that the patients had experienced improvements all areas in which they were impacted, and that any improvement was meaningful for both the patient, and for them. Although some caregivers reported no change (which is expected as the interview population included patients from all treatment groups), a lack of change was still considered to be meaningful as this suggested that, as long as there was no worsening, the patient’s condition was not progressing. Nearly all caregivers reported that they perceived a 1-category change on the CGIS or CGIC items, indicating severity and change respectively, was meaningful to them and the patient.

The unblinded analysis of the exit interview data confirmed that, qualitatively, the experience of the patient as reported by the caregiver was different between the treatment arms, providing qualitative support for the treatment benefit of govorestat when compared to placebo. A larger percentage of caregivers of govorestat treated patients noted improvements in behavior, cognition and motor skills compared to caregivers of patients receiving placebo. In the placebo arms, more caregivers noted no change or worsening. This is consistent with findings observed on clinical outcome measures as part of the clinical trial. Furthermore, the qualitative data from caregivers provide in-depth insights of their unique lived experience that highlight the impact that these improvements have had on the caregiver’s and the patient’s quality of life. The improvements observed led to a reduction on the burden of Classic Galactosemia and may lead to a greater patient’s independence.

The results from the qualitative exit interviews reflect changes in the efficacy endpoints in the AT-007 clinical trial. In particular, this data supports the treatment benefit of govorestat on clinical outcomes compared to placebo, and shows the real-life, positive impact of treatment upon behavior, cognition and motor skills. Full clinical trial findings will be published separately.

### Limitations

All but 3 clinical trial patients who completed Part B of the trial were represented in the interviews analyzed, and so the interview data could be considered truly representative of the clinical trial population. Reflecting the clinical trial population, the interview population was US based, English speaking participants which may limit representativeness of findings. Thus, although cultural differences in the lived experience of Classic Galactosemia are not anticipated, it was not possible to confirm the absence of cultural differences. The interviews were conducted with caregivers, which was considered as appropriate in this pediatric population, and therefore the evaluation of the burden of disease is reported from the caregiver perspective and not directly from patients. Lastly, both blinded and unblinded results are reported here which can introduce the potential for confirmation bias, however to minimize this risk, unblinded analyses were not conducted until full analyses of blinded data and reporting of results were completed and conclusions finalized.

## Conclusions

These exit interviews conducted alongside the AT-007 clinical trial of govorestat in pediatric patients with Classic Galactosemia confirm the burden of disease across multiple domains. The qualitative investigation of the patient experience over the course of the clinical trial suggests that the changes observed are meaningful to the patient and caregiver, and that these changes were more commonly reported in those receiving govorestat compared to placebo. This data therefore supports the treatment benefit of govorestat on clinical outcomes compared to placebo, and shows the real-life, positive impact of treatment upon behavior, cognition and motor skills. The data also confirmed that a 1-category change on the CGIS and CGIC items represents meaningful change. This further demonstrates that the positive treatment effect of govorestat in children with Classic Galactosemia is meaningful and relevant to the patient and the caregiver. Full clinical trial findings will be published separately.

## Data Availability

The datasets used and/or analyzed during the current study are available from the corresponding author on reasonable request.

## Abbreviations

CGIC: Caregiver Global Impression of Change
CGIS: Caregiver Global Impression of Severity
GALT: Galactose-1-phosphate uridylyltransferase
